# Beyond opportunity costs: who bears the implementation costs of reducing emissions from deforestation and degradation?

**DOI:** 10.1007/s11027-016-9736-6

**Published:** 2017-01-20

**Authors:** Cecilia Luttrell, Erin Sills, Riza Aryani, Andini Desita Ekaputri, Maria Febe Evinke

**Affiliations:** 1 Center for International Forestry Research (CIFOR), P.O. Box 0113 BOCBD, Bogor, 16000 Indonesia; 2 NC State University, Raleigh, NC USA; 3 Wildlife Conservation Society, Bogor, Indonesia; 4 Indonesian Institute of Sciences (LIPI), Jakarta, Indonesia; 5 University of Hawai’I, Mānoa, HI USA

**Keywords:** Benefit sharing, Brazil, Cameroon, Costs of climate change mitigation, Costs of REDD+, Indonesia, Opportunity costs of forest conservation, Peru, REDD+, Subnational REDD+, Tanzania, Tropical deforestation

## Abstract

Reducing Emissions from Deforestation and Degradation (REDD+) in developing countries is based on the premise that conserving tropical forests is a cost-effective way to reduce carbon emissions and therefore can be fully funded by international actors with obligations or interests in reducing emissions. However, concerns have repeatedly been raised about whether stakeholders in REDD+ host countries will actually end up bearing the costs of REDD+. Most prior analyses of the costs of REDD+ have focused on the opportunity costs of foregone alternative uses of forest land. We draw on a pan-tropical study of 22 subnational REDD+ initiatives in five countries to explore patterns in implementation costs, including which types of organizations are involved and which are sharing the costs of implementing REDD+. We find that many organizations involved in the implementation of REDD+, particularly at the subnational level and in the public sector, are bearing implementation costs not covered by the budgets of the REDD+ initiatives. To sustain this level of cost-sharing, REDD+ must be designed to deliver local as well as global forest benefits.

## Introduction

Reducing Emissions from Deforestation and Degradation, Plus (REDD+ conservation, sustainable management, and enhancement of forest carbon stocks in developing countries) is intended to be a system of positive incentives for the reduction of deforestation and forest degradation, with countries and sectors historically responsible for carbon emissions paying for the costs of avoiding future emissions from forest loss (Karsenty and Ongolo 2012). A key attraction of REDD+ is the claim that reducing forest emissions is less expensive than reducing emissions from other sectors, and the resulting proposition that the costs of REDD+ can be fully paid by those other sectors (Stern 2006). However, there are persistent concerns about whether the costs will actually be fully covered by carbon payments or will also borne by actors in REDD+ countries (Alston and Andersson 2011). There are two broad areas of concern: the first is that the costs of REDD+, and especially the transaction and implementation costs, are much higher than generally recognized, and the second is that stakeholders in REDD+ countries will not be fairly compensated.

**Fig. 1:**
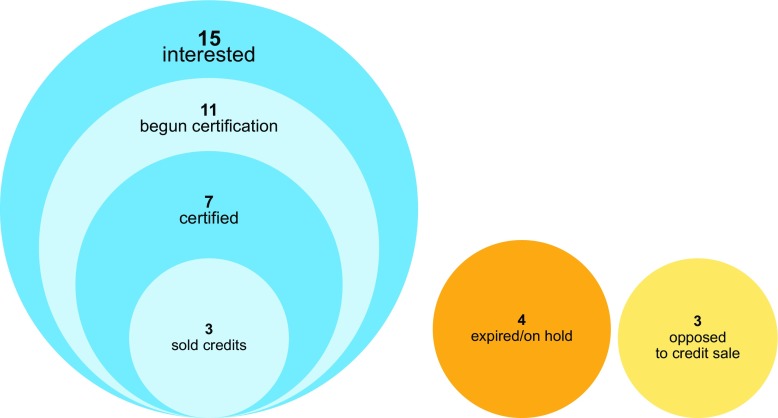
Relationship to the carbon market of REDD+ subnational initiatives in the study sample, as of 2015 (*N* = 22). Seven had either ended or were opposed to selling carbon offset credits. Fifteen were interested in selling credits and had made variable progress, as indicated by the concentric circles

We focus on the intersection of these two areas, examining the incidence of implementation costs across stakeholders from different sectors (public, civil society, and for-profit) operating at different levels (local, regional, national, and international). Specifically, we characterize the incidence of start-up costs of 22 subnational REDD+ initiatives in five countries (Brazil, Peru, Indonesia, Tanzania, and Cameroon). These initiatives reflect the wide variation in REDD+ initiatives across the tropics (Simonet et al. 2014), allowing us to look for empirical regularities and patterns across the range of landscapes and interventions eligible for REDD+. Many—but not all—of these initiatives were designed to generate carbon offset credits for the voluntary market, although only three had actually sold credits by 2015 (Fig. 1). At the time of field research, the 22 initiatives were all in their start-up phase, having defined their intervention areas but not yet begun interventions on the ground. Thus, we characterize them based on their principle objective, their primary source of start-up funding, and their implementation plans (differentiated by the role of forest management), in addition to their relationship with the carbon offset market as shown in Fig. 1.

In the following sections, we first motivate our focus on implementation costs, including how they are defined, concerns that have been raised, and the limited prior research. Based on interviews with key informants familiar with the REDD+ initiatives in our study, we identify and categorize the institutions involved in their implementation, and then look for patterns in the sectors and levels of institutions across countries and across different types of initiatives. Next, we identify the types of institutions that are more or less likely to be fully compensated from the official budgets for the initiatives (e.g., revenues from carbon offsets, or grants for demonstration projects). In essence, we ground-truth concerns about the incidence of REDD+ implementation costs by identifying the types of institutions involved and the types of institutions sharing the costs of implementing these REDD+ initiatives.

## Implementation costs

In this paper, we use the term “implementation costs” to refer to start-up and running costs of administration; institutional arrangements; actions to reduce deforestation or forest degradation (such as patrols); measurement, reporting, and verification (MRV); free prior informed consent (FPIC); and any direct payments or payments for environmental services (PES). These are all activities generally recognized as part of REDD+, although the first three are not always included in the official budgets of REDD+ initiatives. Most are additional to opportunity costs, except for direct payments, which are typically intended as compensation for opportunity costs. Some analysts distinguish between implementation and transactions costs, with the latter being required for participation in the carbon market but not directly reducing deforestation (Dyer and Counsell 2010). Others use the term “institutional costs” for the reforms and capacity building needed to establish REDD+ (Merger et al. 2012). However, these differences are particularly difficult to distinguish in the start-up phase of REDD+, and thus, we group them together as “implementation costs.”

While REDD+ was conceived as an international system of payments to tropical forest countries for reducing forest emissions, critics fear that institutions in REDD+ countries will actually bear many of the implementation costs. For example, Alston and Andersson (2011) discuss the high “hidden costs” of REDD+ that fall heavily on central governments. They suggest that the institutional design of REDD+ is flawed because it depends on host governments to monitor contracts and compliance, and they argue that failure to take the costs of these activities into account is linked to the poor performance of many policies to reduce deforestation. Potvin et al. (2008) provide an example of these costs in Panama, and suggest that the significant costs borne by governments make it difficult to incentivize their involvement. Concerns have also been raised about the degree to which non-governmental organizations (NGOs), and the private sector are subsidizing REDD+ in the short term, potentially creating challenges for long-term commitment (O’Sullivan et al. 2010; Climate Investment Fund 2013).

These concerns are linked to more fundamental criticisms of REDD+ from a vocal and highly visible opposition that operates under taglines such as “No REDD” or “REDD Alert” (Cabello and Gilbertson 2010). These opponents warn that powerful actors with vested interests in carbon markets will capture most carbon revenues, leaving underfunded institutions to bear the costs of implementation and vulnerable stakeholder groups to bear the opportunity costs (e.g., Gilbertson 2011). Most research on the costs of REDD+ has focused on opportunity costs (e.g., Wertz-Kanounnikoff 2008; Pagiola and Bosquet 2009; Fosci 2013) including their distribution across stakeholders (e.g., Adams et al. 2010; White et al. 2011; Delacote et al. 2014), to the exclusion of implementation costs. For example, in their review of 92 studies that provide estimates of REDD+ costs and benefits, Rakatama et al. (2016) found that only 21 reported on implementation and transaction costs. This is consistent with the lack of attention to these costs in early discussions about REDD+ (Eliasch 2008, McKinsey and Company 2009, UNEP 2011).

There is increasing recognition of the importance of implementation and transactions costs (Fisher et al. 2011; Fosci 2013; McCann 2013), especially in the start-up phase (Wertz-Kanounnikoff 2008; Phan et al. 2014) and in small-scale projects (Mooney et al. 2004; Galik et al. 2009). For example, Merger et al. (2012) and Rendon Thompson et al. (2013) found that these costs were significant in REDD+ projects in Tanzania and Peru, respectively. However, there has been less attention to the distribution of these costs across stakeholders (Rakatama et al. 2016). We help fill this gap by characterizing the types of institutions incurring these costs for subnational REDD+ initiatives and by exploring whether these costs are recognized and covered by the official budgets of the initiatives.

## Methods

We consider the start-up phase of 22 initiatives (six in Indonesia, six in Tanzania, five in Brazil, three in Cameroon, and two in Peru) included in CIFOR’s Global Comparative Study on REDD+ (CIFOR-GCS) sample^1^ (Table 1). CIFOR-GCS selected these initiatives based on five criteria: (i) they conform to an operational definition of the term “REDD+” as actions whose primary aim is to reduce emissions from deforestation and forest degradation and/or to increase removals through conservation, sustainable management, and enhancement of the carbon stocks of existing forest in developing countries; (ii) they intend to monitor, report, and/or transact reductions in carbon emissions or increases in removals (in a quantified manner); (iii) they had defined site boundaries and identified intervention villages before the baseline GCS survey; (iv) they planned to offer conditional incentives to achieve reductions in deforestation and degradation; and (v) they did not plan to offer those incentives until after the baseline GCS survey (Sunderlin et al. 2016). By comparison to an independently compiled catalog of REDD+ projects (Simonet et al. 2014), Sunderlin et al. (2016) confirms that the average initiative in the GCS sample is similar to the average REDD+ initiative in each of the five study countries.

**Table 1:** Key characteristics of 22 subnational REDD+ initiatives (in Brazil, Cameroon, Indonesia, Peru, and Tanzania)

Initiative	Size^a^	Primary proponent type	Primary objective	Primary funding source	Degree of importance of SFM	Intent and realization of carbon sales
Brazil						
Acre	Large	Government	Co-benefits (conservation, biodiversity and SFM)	Government	SFM activities planned or minor	Certified
Cotriguaçu	Medium	Civil society-government	Reduce carbon emissions in order to obtain carbon funding or sell carbon credits	Government	SFM activities planned or minor	Opposed to credit sale
Jari/Amapá	Small	For-profit	Reduce carbon emissions in order to obtain carbon funding or sell carbon credits	NGO	SFM certification a central objective	Selling credits
SFX	Large	Civil society-government	Demonstrate viability of REDD+ by reducing carbon emissions	Government	SFM important part of strategy	Opposed to credit sale
Transamazon	Small	Civil society	Demonstrate viability of REDD+ by reducing carbon emissions	Government	Aim to stop illegal logging only	Opposed to credit sale
Cameroon						
Mt. Cameroon	Small	Government	Demonstrate viability of REDD+ by reducing carbon emissions	Private	Aim to stop illegal logging only	Not yet certifying
SE Cameroon (East)	Small	Civil society	Co-benefits (conservation, biodiversity and SFM)	International/national donor	Aim to stop illegal logging only	Certified
SE Cameroon (South)	Small	Civil society	Co-benefits (conservation, biodiversity and SFM)	International/national donor	Aim to stop illegal logging only	Certified
Indonesia						
Katingan	Medium	For-profit	Reduce carbon emissions in order to obtain carbon funding or sell carbon credits	Private	SFM important part of strategy	Certification in progress
KCCP	Small	Civil society	Co-benefits (conservation, biodiversity and SFM)	NGO	SFM activities planned or minor	Certification in progress
KFCP	Medium	Government	Demonstrate viability of REDD+ by reducing carbon emissions	International/national donor	SFM important part of strategy	Expired/on hold
Rimba Raya	Small	For-profit	Co-benefits (conservation, biodiversity and SFM)	Private	Aim to stop illegal logging only	Selling credits
TNC within BFCP	Large	Civil society	Reduce carbon emissions in order to obtain carbon funding or sell carbon credits	International/national donor	SFM certification a central objective	Not yet certifying
Ulu Masen	Medium	Government	Co-benefits (poverty reduction and community development)	Private	SFM activities planned or minor	Expired/on hold
Peru						
Madre de Dios	Medium	For-profit	Co-benefits (conservation, biodiversity and SFM)	NGO	SFM certification a central objective	Selling credits
Ucayali	Medium	Civil society	Reduce carbon emissions in order to obtain carbon funding or sell carbon credits	International/national donor	SFM important part of strategy	Certified
Tanzania						
Kigoma	Small	Civil society	Reduce carbon emissions in order to obtain carbon funding or sell carbon credits	International/national donor	SFM important part of strategy	Expired/on hold
Kilosa	Medium	Civil society	Demonstrate viability of REDD+ by reducing carbon emissions	International/national donor	SFM activities planned or minor	Not yet certifying
Lindi	Small	Civil society	Demonstrate viability of REDD+ by reducing carbon emissions	International/national donor	SFM activities planned or minor	Certification in progress
Mpingo	Medium	Civil society	Reduce carbon emissions in order to obtain carbon funding or sell carbon credits	International/national donor	SFM important part of strategy	Certification in progress
Shinyanga	Small	Civil society	Reduce carbon emissions in order to obtain carbon funding or sell carbon credits	International/national donor	SFM important part of strategy	Certification in progress
Zanzibar	Small	Civil society	Demonstrate viability of REDD+ by reducing carbon emissions	International/national donor	SFM certification a central objective	Expired/on hold

^a^Size is defined as an intervention area <1000 HA (small), 1000–20,000 HA (medium), or >20,000 HA (large)

As described in Sunderlin et al. (2016), baseline data for CIFOR-GCS were collected between 2010 and 2013, before interventions began, and follow-up data were collected in 2014. A lead field researcher was assigned to each site and became familiar with the local context and institutions during weeks spent in the field. This researcher was responsible for (i) structured village and household surveys, (ii) semi-structured interviews with key informants, and (iii) desk review of documentation and maps. As part of the baseline data collection, researchers interviewed representatives of the lead implementing organizations of the initiatives, as well as key informants from other stakeholder institutions (interview guides available from Sunderlin et al. (2010)). The researchers drew on both these key informant interviews and written sources to generate complete lists of the organizations involved in each initiative and to characterize them as either cost-sharing or being compensated through the REDD+ budget. For our analysis, we use the characteristics of each initiative and each organization as determined by the lead researchers in each site.

For each of these initiatives, the researchers generated lists of all organizations that had incurred “significant” costs in the start-up phase of REDD+, including for design, planning, preparation, or oversight. Significant is defined as having spent, or having control over, at least (a) 5% of the total budget of the initiative to date, (b) at least 1 month of person-days in the start-up phase, or (c) at least five person-days per month in continuing or recurrent costs or equivalent financial outlay. The organizations were then categorized into three groups:

1. Those that have all of their REDD+ related costs covered by the official REDD+ budget, including possibly earning a surplus from REDD+ (e.g., using MRV resources to also monitor biodiversity in the region where the REDD+ initiative is located)
2. Those that are burden-sharing—incurring more costs for the initiative than their portion of the official budget, i.e., providing significant financial, labor, or other in-kind cost-sharing
3. Those that both have their costs covered and are burden-sharing in different roles and modes of input

## Characterizing the REDD+ initiatives

The 22 initiatives listed in Table 1 are of very different sizes and institutional arrangements. Half cover less than 1000 ha each, while the other half range in size up to 157,490 km^2^. Thirteen initiatives are led by NGOs (civil society), four by companies (for-profit), three by government organizations, and two jointly by civil society and government organizations. In addition to these lead proponents, many other organizations—often from different sectors—have been involved in the start-up phase of these initiatives, as discussed below.

We further categorized the initiatives according to their objectives and implementation plans. Specifically, we considered four dimensions that vary substantially across initiatives (Table 1):

1. The proponent’s stated primary objective
2. The role of sustainable forest management in the implementation plan
3. The primary source of start-up funding
4. Intentions and realization of carbon credits sales (Fig. 1)

We assessed REDD+ implementation strategies based on the primary objective of each initiative, as identified by its lead proponent. We categorized the initiatives into those whose proponents are *primarily* seeking to

1. Reduce carbon emissions in order sell offset credits (i.e., implement REDD+) (eight)
2. Demonstrate the viability of REDD+ (seven)
3. Generate non-carbon benefits (e.g., conservation, biodiversity, sustainable forest management, or poverty reduction) (seven)

Regardless of the proponents’ primary objectives, they all intend to reduce forest clearing for agriculture and/or livestock. However, the initiatives differ in terms of the degree to which sustainable forest management (SFM) for timber and non-timber products is incorporated into their implementation plans. SFM certification (e.g., through the Forest Stewardship Council) is a core element of the implementation strategies of four initiatives, based on the assumption that certified forests are more valuable, and therefore less susceptible to deforestation. Seven initiatives have SFM as an important but not core element of their strategies. The proponents of six initiatives reported that SFM is a minor or planned future activity. Finally, five of the initiatives seek to prevent illegal logging, but do not otherwise promote SFM. It is notable that all eight initiatives with the primary objective of selling offset credits have SFM as a core or important part of their strategy.

Half (11) of the initiatives in our sample were initially funded primarily by donor sources, while the remaining initiatives were funded primarily by NGOs (three initiatives), private companies (four initiatives), or national governments (four initiatives, all in Brazil) (Table 1). This distribution reflects the “aidification” of REDD+, with most funding coming from public sources, including Official Development Assistance (ODA) and government donors (Streck and Parker 2012; Seymour and Angelsen 2012; Angelsen 2013). Two out of four government-funded initiatives, but only four out of 11 donor-funded initiatives, stated that demonstrating the viability of REDD+ was their primary objective.

The initiatives in our sample vary widely in terms of their relationship with the carbon offset market. Figure 1 shows that while most initiatives are interested in selling credits, they had made variable progress by the beginning of 2015, falling into the following six categories:

1. Three initiatives had already sold credits in a voluntary or subnational market
2. Four initiatives had been certified to sell voluntary carbon offsets (e.g., by the Climate Community and Biodiversity Alliance or by Plan Vivo) but had not yet made any sales
3. Four initiatives had completed at least one step towards certification (e.g., developing and submitting a methodology), but had not yet achieved certification or sold credits
4. Four initiatives had not made any progress towards selling credits, although they remain interested in supplying carbon offsets
5. Three initiatives had decided (either initially or at a later stage) that they are philosophically or strategically opposed to selling credits in voluntary or subnational markets
6. Four initiatives had expired or been put “on hold” as of the beginning of 2015

The second and third categories suggest but do not prove interest in selling carbon credits. All of the initiatives with non-carbon benefits as their primary objective have been certified, or are making progress towards certification (e.g., they have a project development document). Proponents might seek certification in order to establish their legitimacy and bolster their case for external support from any source—not just the carbon market. The last three categories, which include 11 out of 22 initiatives, are the least successful according to Reynolds’ (2012) definition of a successful REDD+ project as one that (i) does not collapse during the study period and (ii) sells carbon offsets. However, we distinguish between initiatives that are opposed to selling carbon credits (category 5) and therefore cannot be judged as less successful because of this, and those that are interested but have not made progress towards sales (category 4).

## Results

### Initiatives by implementing organizations

We found wide variation in the number of organizations involved in the start-up phase of the different initiatives. The Brazilian and Indonesian initiatives that cover entire jurisdictions involve the most organizations, but there are also a large number involved in a donor-funded demonstration project in Indonesia (the Kalimantan Forests and Climate Partnership or KFCP).

As shown in Table 2, international organizations played a significant role in the implementation of all but five of the initiatives, and national and subnational organizations were involved in all but four (organizations that operate in an area larger than a single village but smaller than an entire country are considered subnational). In contrast, there were no local (village) level organizations significantly involved in the start-up phase of more than half (13 out of 22) of the initiatives.

**Table 2:**
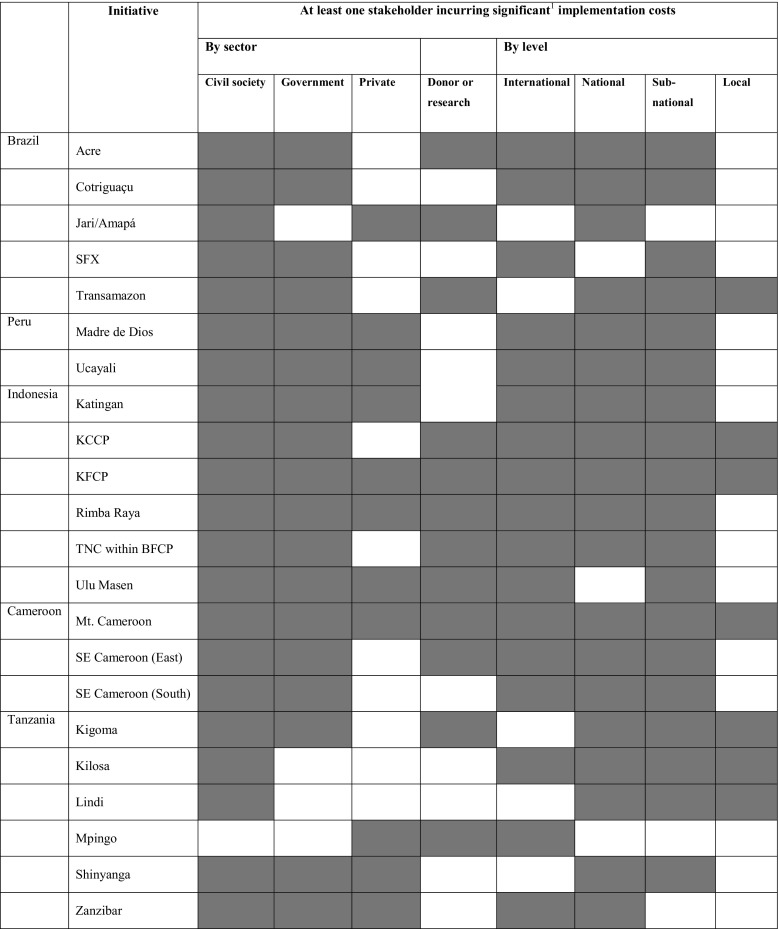
Involvement of organizations from different sectors and operating at different levels in 22 subnational REDD+ initiatives

^a^“Significant” costs are at least 5% of the total project budget, or at least 1 month of person-days in start-up phase, or at least five person-days per month on-going costs. Shaded cells indicate that at least one stakeholder of a given type or level incurred significant cost for implementation of a given initiative in its start-up phase

Civil society organizations were involved in the start-up phase of all initiatives except one, while government agencies were involved in 14 initiatives, and private sector organizations were involved in 11 initiatives. Table 2 suggests three major categories of initiatives based on the sectors involved in their start-up phase, i.e., those implemented by

1. Civil society and government (eight initiatives)
2. Civil society and for-profit sector (five initiatives)
3. All three sectors (five initiatives)

There is an apparent negative relationship (not statistically significant in our sample of 22 initiatives) between the importance of forest management in the strategy for a REDD+ initiative and the involvement of civil society institutions. Specifically, the larger the percentage of civil society institutions involved in an initiative, the less focus on sustainable forest management. Sixty-four percent of organizations that do not promote SFM (except to prevent illegal logging) are from civil society, compared to 35% of the organizations in initiatives that have certification as a core element of their strategy. On the other hand, the percentage of implementing organizations that are for-profit increases with the initiative’s degree of attention to SFM (from 11% in initiatives that do not actively promote SFM to 27% in initiatives that are pursuing certification).

We also note a possible relationship between an initiative’s approach to the carbon credit market and involvement of the private sector in its implementation. Across initiatives that have sold carbon credits, more than a third (37%) of the implementing organizations are for-profit, and no government institutions are involved. In contrast, initiatives opposed to credit sales had no private sector involvement. Across the four initiatives that had expired by the beginning of 2015, a third of implementing organizations were public sector, and only a fifth (21%) were from the private sector.

### Implementing organizations by sector and level

**Fig. 2:**
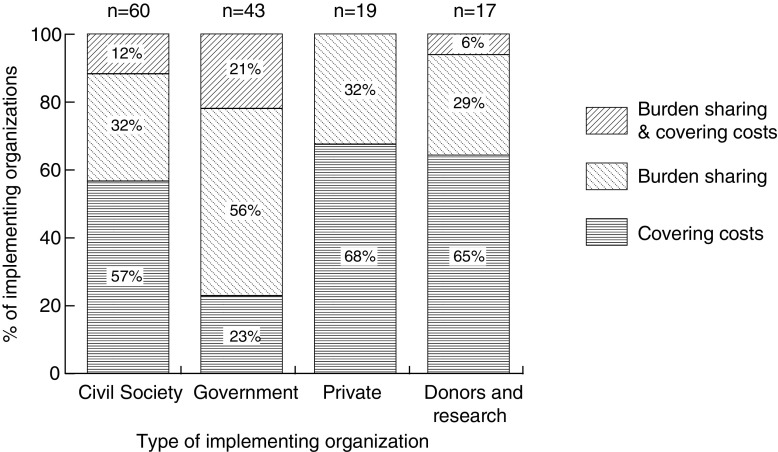
The percentage of REDD+ implementing organizations from each sector that were “burden-sharing” (cost sharing implementation of the initiative) vs. covering their costs (with all costs incurred covered by the budget for the initiative) in the start-up phase of subnational REDD+ initiatives (*N* = 139)

The 139 organizations involved in the start-up phase of the 22 initiatives in our sample provide a picture of who is involved in the implementation of REDD+ on the ground (counts given in Fig. 2). There are only 19 for-profit organizations participating across all 22 initiatives, including private investors, firms providing management or technical services such as carbon accounting, financing, and forestry expertise, and companies operating in the carbon offset market. Civil society organizations are the most frequently occurring (60/139) and government the second most frequently occurring (43/139). In addition to organizations from the public, private, and civil society sectors, we identify a small number (17) of organizations that are best characterized as donors or research institutions, such as universities.

**Fig. 3:**
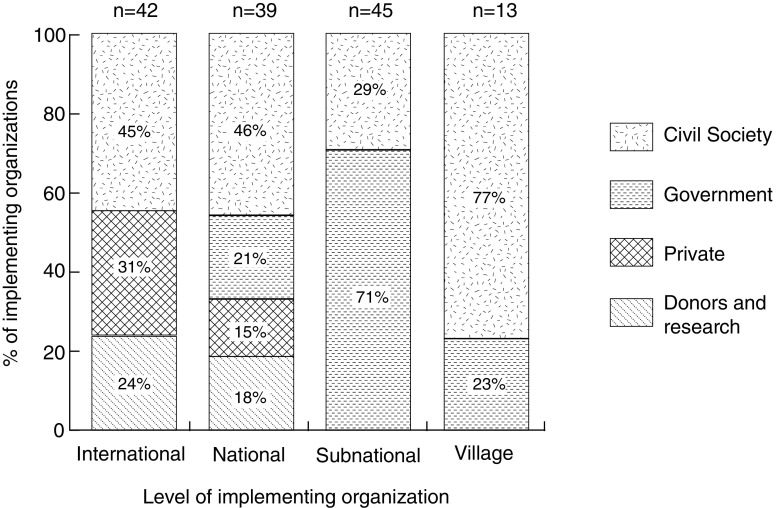
The percentage of REDD+ implementing organizations operating at each level (international, national, subnational, and local) that are in each sector (public, civil society, private, and donors/research) (*N* = 139)

Considered by level, the largest numbers of organizations involved in the start-up phase of initiatives operate at the subnational (45 out of 139) and international (42 out of 139) levels (Fig. 3). When we compare the level and sector of these organizations, we find that most subnational organizations (71%) are government agencies, whereas most of the international organizations are from civil society. Private sector organizations are either national or international. The few village level organizations (*n* = 13 across all initiatives) are mostly from civil society (77%). Subnational organizations are most prevalent (50%) among initiatives funded primarily by the government, and national organizations are most prevalent among initiatives funded primarily by NGOs. Three of the four initiatives that lacked national level organizations had expired as of 2015. It is also interesting to note that initiatives funded primarily by government sources involve more subnational and for-profit organizations. This suggests that government funding for initiatives may be associated with broader participation, including private sector and subnational institutions.

### Patterns of burden and benefit-sharing across types and levels of organizations

**Fig. 4:**
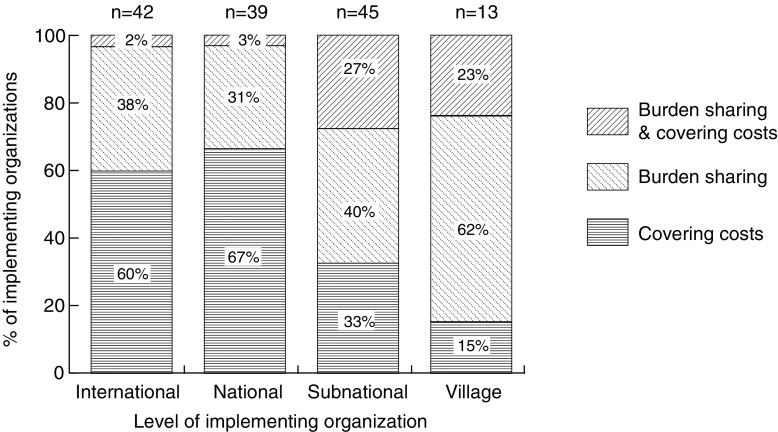
The percentage of REDD+ implementing organizations operating at each level (international, national, subnational, and local) that were “burden-sharing” (cost sharing implementation of the initiative) vs. covering their costs (with all costs incurred covered by the budget for the initiative) in the start-up phase of subnational REDD+ initiatives (*N* = 139)

To assess the concern that the costs of REDD+ will fall on institutions in REDD+ countries, we assess which types of organizations are more likely to (i) have their costs fully covered (or possibly more than fully covered) by the initiative budget, and which are more likely to (ii) share the financial burden of implementing the initiative.^2^ A high percentage of village and subnational level organizations are burden-sharing (62 and 40%, respectively), while 60 and 67% (respectively) of international and national institutions cover their costs (Fig. 4). Comparing across sectors, we find that burden sharing is most common among government (56%) (Fig. 2). Thus, we find some basis for concerns that REDD+ country institutions will end up shouldering part of the cost of REDD+, at least in the start-up phase.

**Fig. 5:**
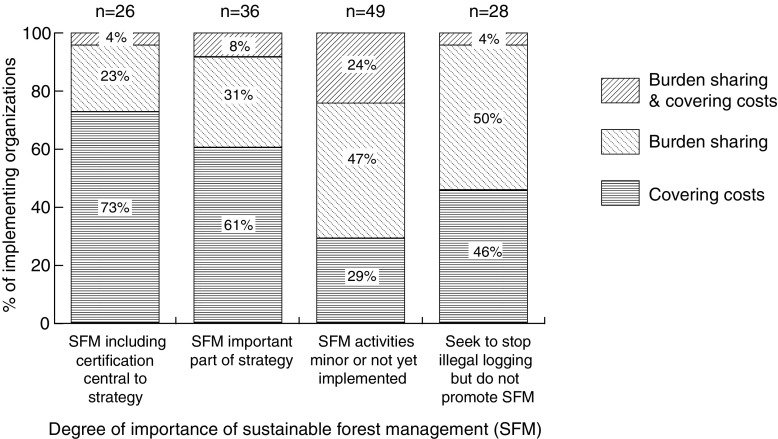
The percentage of REDD+ implementing organizations involved in initiatives with different ways of incorporating sustainable forest management (SFM) that were “burden-sharing” (cost sharing implementation of the initiative) vs. covering their costs (with all costs incurred covered by the budget for the initiative) in the start-up phase of subnational REDD+ initiatives (*N* = 139)

Initiatives funded primarily by governments in their start-up phase involve a much higher percentage of burden-sharing institutions (56%) compared to initiatives funded primarily by NGOs (16%). Thirty-four percent of institutions involved in donor funded initiatives and 46% of institutions involved in private sector funded projects are burden-sharing. At least in our sample, donor and NGO funded initiatives are most likely to balance compensation with actual costs incurred from the beginning of the initiative, whereas the government funded initiatives are more likely to involve organizations that do not fully recover their costs, at least during the start-up phase. However, this could be due to the fact that all government funded initiatives in this sample occur in Brazil and three out of four of these initiatives are opposed to credit sales (Table 1). Initiatives with lead proponents opposed to sale of carbon credits have the highest percentage (50%) of organizations that are burden-sharing. Thus, opposition to credit sales is accompanied by willingness to share the costs of REDD+, as might be expected. Another pattern is that initiatives with a stronger focus on SFM have a higher percentage of organizations that have their costs fully covered, e.g., 73% of organizations involved in initiatives that focus on forest certification cover their costs (Fig. 5).

## Discussion

Our examination of the organizations involved in and sharing the costs of implementing REDD+ leads to several key insights discussed here. This further leads us to explore the motivations of these organizations for becoming involved in REDD+.

### Involving more organizations does not necessarily increase the cost of REDD+

There are 139 organizations are involved in the implementation of the 22 subnational REDD+ initiatives in our sample. Based on a review of carbon forestry projects, Milne (1999) suggests that involving a large number of parties in project implementation increases implementation costs. This raises the question of why there are so many organizations involved in REDD+ initiatives. In our data, there is a weak, but positive, correlation between the number of implementing organizations and the proportion of those organizations that are burden-sharing (i.e., cost-sharing the implementation of REDD+) in any given initiative. That is, the more organizations involved, the more likely there is to be cost-sharing. Alston and Andersson (2011) suggest that the costs of REDD+ can be reduced by encouraging a variety of organizations to take on related tasks. Adding organizations can also help reduce total costs if they have complementary expertise (e.g., staff with different backgrounds and training). Furthermore, they may be able to capture additional resources available only to certain types or levels of organizations. Agrawal et al. (2011) suggest that efforts to promote complementarity of interests and capacities among different actors “may help achieve multiple objectives that REDD+ efforts have come to symbolize for different stakeholders.”

### REDD+ initiatives have been subsidized through government cost-sharing

Start-up of the REDD+ initiatives that we studied has been supported by numerous government institutions. In particular, many government entities bear non-monetary transactions costs, such as staff time, which have also been identified as important hidden costs in community forestry (Schreckenberg and Luttrell 2009). Government subsidization is more apparent in Brazilian initiatives and less common among Indonesian initiatives, both because there are more government institutions involved in Brazil and because many of those institutions are cost-sharing. The willingness of both national and subnational governments to bear some of the costs of establishing initiatives suggests that their goals go beyond immediate cost-recovery. For example, governments may seek to use the early initiatives to build readiness and support for REDD+, or to generate non-carbon co-benefits. An important area for further research is whether these public investments in REDD+ are “crowding out” or “crowding in” investments by the private sector and civil society (cf. Andreoni and Payne 2003; Albers et al. 2008).

### Challenging the notion of REDD+ as a centralizing force: subnational organizations play a significant role

A diverse range of stakeholder institutions, across all governance levels, were involved in the start-up phases of most of the initiatives studied. Subnational and international organizations are particularly numerous. The burden on subnational institutions was predicted by Nepstad et al. (2007), who highlighted the costs that the federal and state governments in Brazil were likely to incur to reduce emissions. Brazilian initiatives are supported by a particularly large number of subnational organizations: all but one of the Brazilian initiatives that we studied have more subnational than national or international organizations significantly involved in implementation. This is related to on-going decentralization of environmental governance in Brazil, including federal efforts to hold local governments responsible for slowing deforestation, e.g., through a “blacklist” or “embargo” of municipalities that deforest the most (Assuncao and Rocha 2014; Cisneros et al. 2015). Our data also reveal a large number of subnational actors involved in Indonesian initiatives, in contrast to arguments that this level has been excluded from REDD+ policy discussions in Indonesia (Gallemore et al. 2015). Phelps et al. (2010) also warned that REDD+ could encourage recentralization of forest management across the tropics, due the requirements of performance-based financing. However, our data suggest that even if *policy* discussions are dominated by national level organizations, organizations at the subnational level are highly involved in implementation. Indeed, four of the initiatives in our sample (in Tanzania and Brazil) had no significant involvement of national organizations.

Some observers have argued that in response to the substantial costs of REDD+, a polycentric (or multileveled) system is emerging (Ostrom 2010, 2012). They argue that multilayered, collective action problems need global institutions to channel finance, but local institutions to monitor management and distribute benefits (Corbera and Brown 2008). Ideal polycentric systems benefit from the complementary strengths of multiple institutions (Andersson and Ostrom 2008; Nagendra and Ostrom 2008; Agrawal et al. 2011), keeping transaction costs low (Alston and Andersson 2011; Leifeld and Schneider 2012). The variety of levels and types of implementing organizations in our sample is consistent with a polycentric system (Ostrom 2010, 2012). Reynolds (2012) examines variation in local, subnational, and national level institutions and finds that working across levels can make carbon forestry projects more likely to succeed. This is echoed in our finding that the initiatives that have expired did not have implementing organizations at all levels and from all sectors, with gaps especially in national level and private sector representation.

While the initiatives in our sample involved organizations from a variety of sectors and levels, there is one notable gap: in many (13 out of 22) initiatives, particularly in Brazil, Peru, and Indonesia, there were no village level institutions involved in the start-up phase, or in other words, the core of the polycentric governance system was hollow. The exception was Tanzania, where, in most initiatives, village level organizations were most common. This may reflect the way in which Norwegian funding for REDD+ in Tanzania specifically targeted local capacity building, in order to move forward while national institutions and rules for REDD+ were still being developed (Rantala et al. 2015). In the short-run, the hollow core does not appear to have been a major stumbling block for initiatives: there were no village organizations and relatively few subnational organizations involved in implementation of initiatives that were certified and that sold credits (one definition of “success”).

### Motivation for involvement in REDD+ goes beyond cost-recovery or profit

Cost compensation is a prominent part of the discourse on REDD+ benefit-sharing (Luttrell et al. 2013). A commonly held view is that international funding for REDD+ should cover both the opportunity costs of landowners and the implementation costs of the organizations involved, thus matching benefits to the costs incurred. Failure to cover costs could lead to a motivation deficit, and thus a sustainability problem. However, we find that not all organizations behave according to this “cost compensation” logic in the start-up phase of initiatives. For example, many (40%) subnational organizations supporting the implementation of initiatives are bearing the costs themselves (Fig. 4). This presents a stark contrast to predictions that funding for REDD+ would be diverted to support conservation efforts and other national and local priorities (Pagiola and Bosquet 2009; Harvey et al. 2010; Phelps et al. 2011). Rather than siphoning off REDD+ funds to support related activities, the organizations involved from both the public sector and civil society are sharing the costs of implementation.

This willingness to share the burden perhaps reflects a strategy of getting REDD+ up and running, with the hope that it will eventually generate a surplus. Specifically, governments may be willing to use public funds to reduce emissions, with the hope that their investment will leverage other investment or REDD+ funds that can be shared as benefits (e.g., for forest stewards). A second possible explanation is that governments (and local organizations) have the greatest interest in securing non-carbon co-benefits from REDD+ and are thus willing to invest in its establishment. Two of the four initiatives funded primarily by private sources indicated that non-carbon benefits were their primary objective. The proponent of one of these (Ulu Masen) stated that its primary objective was poverty reduction, while the proponent of Rimba Raya stated that its primary objectives were conservation, biodiversity, and SFM (Table 1). Both of these initiatives aimed to sell carbon offsets and thus may have been interested in generating co-benefits partly to secure access to the voluntary carbon market. Dixon and Challies (2015) also find a range of motivations among private sector investors in REDD+ in Indonesia. They suggest that those who seemed to be willing to derive little or no immediate financial return were the most active in REDD+, while those who prioritized financial profit had typically suspended or slowed their activities or spread their risk across other investments.

A third possibility is that host governments view investment in REDD+ as part of voluntary national contributions to climate change mitigation (i.e., nationally appropriate mitigation actions (NAMAs) and nationally determined contributions (NDCs)) (Boos et al. 2015; Fridahl et al. 2015). This could turn REDD+ into an unfunded mandate for subnational governments. Alternatively, it could be that government institutions are willing to share the costs in order to obtain more influence and control over implementation, thereby avoiding the paternalistic relationships associated with international financing (Sanginga et al. 2007).

## Conclusions: implications for REDD+

Our findings, based on the experiences of 22 subnational REDD+ initiatives in five countries across the tropics, provide evidence on participation and cost-sharing in REDD+. This is globally important because tropical deforestation has accounted for over 10% of global carbon emissions (Tyukavina et al. 2015), and many consider REDD+ to be a mitigation strategy which is “essential to meeting the <2 degree threshold that the international community has adopted” (Zarin et al. 2016). An important contribution of our study is to make REDD+ more transparent by characterizing the organizations involved in the implementation of different types of subnational initiatives. Recent years have seen a shift of the focus of mitigation policy to the subnational level as subnational approaches as increasingly recognized as critical for the implementation of the global climate deal and INDCs (Swette et al. 2015). While more than a third of the initiatives in our study were designed to generate carbon credits for sale, others were intended to demonstrate REDD+ or to generate co-benefits. The experiences of this varied group of initiatives provides important lessons for REDD+ as an international effort to mitigate climate change.

We confirm the existence of a polycentric system for implementing REDD+, with 139 institutions from different sectors and levels involved in implementation of the 22 initiatives that we study. Many public and civil society organizations in tropical forest countries are sharing the cost of getting REDD+ up and running. Subnational institutions play a far larger role than is often assumed, and they have been willing to subsidize REDD+ initiatives by cost-sharing both staff time and expenses. Initiatives implemented by a narrower range of stakeholders in terms of both sectors (i.e., not including private sector) and levels (i.e., not including national) are more likely to have expired. While this suggests that a robust polycentric system is emerging, we also found evidence of a hollow core, with very few local organizations involved in implementation of subnational initiatives in most countries that we studied. This avoids burdening local institutions with implementation costs, but at the same time, it limits their participation and voice in the implementation of REDD+.

Future research and policy decisions about the design of REDD+ as a global mitigation strategy should give greater attention to the subnational level, seeking to understand both the motivations and the incentives necessary to maintain the interest of subnational organizations involved in the implementation of REDD+ (cf. Swette et al. 2015). In the 22 initiatives that we studied, we found that subnational governments were particularly likely to bear part of the financial burden of getting REDD+ established. This points to the importance of understanding and reinforcing their motivations for involvement in REDD+, which could range from expectations of future carbon revenues to recognition of local co-benefits. Researchers and architects of REDD+ should also pay careful attention to the local level, including the reasons for and implications of limited involvement by local organizations.

While REDD+ was originally promoted as a quick and easy global mitigation strategy (just requiring that people not cut down trees), the challenges of on-the-ground implementation are increasingly recognized. The experience of the subnational REDD+ initiatives considered here suggests that getting REDD+ established will likely involve numerous institutions, especially from civil society and the government, and from the subnational and national levels. To be effective as a global mitigation strategy, REDD+ must be designed to keep those institutions engaged, either by making the promised international transfers or by making transparent and acknowledging the multiple local and regional co-benefits of conserving forest.

These findings are also relevant to broader landscape and jurisdictional approaches to low carbon development and voluntary national contributions to climate change mitigation. Perhaps even more than REDD+, these approaches are likely to rely on the engagement of a wide range of stakeholders and institutions. To sustain their involvement and design benefit sharing systems, it is important to map out the involvement of organizations from different sectors and operating at different levels and recognize which are effectively subsidizing mitigation through the cost-sharing of implementation.

## Footnotes

1 The initiatives selected for the CIFOR-GCS sample are described in Sills et al. (2014). Due to incomplete stakeholder information, we discarded two initiatives from our sample (one in Brazil and one in Vietnam). Because we differentiate between distinct sites with different groups of stakeholders, we sub-divided one initiative in Cameroon and one initiative in Tanzania.

2 Some stakeholder institutions were described as both “burden-sharing” and as “benefitting more than the costs incurred,” and a judgment could not be made as to which of these was dominant. We do not include these institutions in any of the figures reported for either “burden-sharing” or “covering costs.”
